# A process-oriented functional capacity assessment: First application of the neuropsychological evaluation of the UPSA (NEUPSA)

**DOI:** 10.1080/23279095.2026.2676165

**Published:** 2026-05-31

**Authors:** Alannah Miranda, Breanna M. Holloway, Elizabeth Peek, Holden Rosberg, Karli Raven, Elizabeth W. Twamley, Arpi Minassian, William Perry

**Affiliations:** a Department of Psychiatry, University of California, San Diego, California, USA;; b Department of Neuroscience, University of California, San Diego, California, USA;; c VA San Diego Healthcare System, VA Center of Excellence for Stress and Mental Health, San Diego, California, USA

**Keywords:** Bipolar disorder, cannabis, cognition, functional assessment

## Abstract

In this study we describe the Neuropsychological Evaluation of the UPSA (NEUPSA), a structured scoring system of the *UCSD Performance-based Skills Assessment-2* (UPSA-2) aimed at capturing the cognitive processes that underlie functional capacity. We sought to apply the NEUPSA in both healthy and clinical populations (psychiatric disease, substance use), compare NEUPSA scoring against standardized cognitive test performance and test whether the NEUPSA was sensitive to effects of cannabis use and bipolar disorder (BD) on neuropsychological functioning. Eighty-eight participants from a larger study on the cognitive effects of cannabis use and BD were administered the UPSA-2. During the administration of the UPSA-2, participant behavior was assessed and scored in five cognitive domains (memory, attention, inhibition/impulsivity, speed of information processing, executive function) using the NEUPSA scoring system. Participants were also administered validated computerized cognitive tests, including the Iowa Gambling Task (IGT; executive functioning) and the 5 Choice-Continuous Performance Task (5C-CPT; attention, speed of information processing and inhibitory control). Performance in the NEUPSA was significantly correlated with performance in the IGT and 5C-CPT for some cognitive domains. Performance on the NEUPSA differed among groups with BD and cannabis use, with healthy comparison participants generally performing better than clinical groups. This pilot study highlights the potential of the NEUPSA as a novel tool for evaluating cognition within performance-based functional assessments. Further refinement and validation of the NEUPSA is necessary and could serve as a valuable resource in both clinical and research settings, advancing our understanding and treatment of cognitive deficits in clinical populations.

## Introduction

Accurate assessment of functioning (i.e., ability to engage in vital tasks of daily living) is critical for the diagnosis and treatment of psychiatric conditions. Instruments used to evaluate functional deficits in psychiatric populations, such as the Global Assessment of Functioning Scale (GAF), rely on self-report and survey tools (Aas et al., 2018; Lin et al., 2026; Martino et al., 2011; Sanchez-Moreno et al., 2009). By contrast, the University of California San Diego (UCSD) Performance-Based Skills Assessment (UPSA (Patterson et al., 2001)), is specifically designed to assess functional capacity through role-play tasks that simulate real-world activities such as financial management, transportation, and medical appointments. Unlike self-report questionnaires or clinician-rated scales, the UPSA directly measures performance, offering a more objective index of functional capacity. In its original validation, the UPSA was applied to middle-to-older aged adults with severe mental illness, who performed significantly worse than healthy comparisons (Patterson et al., 2001). UPSA performance also correlated strongly with the Direct Assessment of Functional Status (DAFS), supporting its validity as a measure of functional capacity.

Although the UPSA assesses functional capacity, it does not formally capture how a person approaches each task, which can offer a deeper insight into the cognitive underpinnings of functional performance. Role-play-based assessments, such as the UPSA, allow administrators to observe naturalistic problem-solving strategies, error patterns, and behavioral regulation. Such observations align with the process-oriented approach (Kaplan and colleagues), which is a neuropsychological framework that analyzes cognitive efficiency, errors, and behavioral strategies rather than merely recording a score (Kaplan, 1988). The process approach provides a richer clinical picture by identifying which cognitive domains, such as attention, executive function, or processing speed, contribute to observed performance deficits.

With the process approach in mind, we aimed to quantify the component cognitive processes that underlie successful performance on UPSA tasks, an approach that has been utilized for other neuropsychological assessments (Kaplan, 1995, 1999; Lamberty & Axelrod, 2006; Minassian et al., 2003). In this pilot study, we developed the Neuropsychological Evaluation of the UPSA (NEUPSA), a structured scoring system applied in parallel with the UPSA. Specifically, the NEUPSA provides a framework to organize and quantify behavioral observations from the UPSA based on cognitive domain, namely: memory, attention, inhibition/impulsivity, speed of information processing, and executive function. Through this approach, the NEUPSA combines the real-world relevance of the UPSA with quantification of cognitive functions in a single session.

Here we present the first application of the NEUPSA, treated as a single illustrative case during which the tool was used with a mixed cohort of healthy individuals and people with bipolar disorder (BD), with and without regular cannabis use, who were part of a larger study investigating the effects of cannabis use and BD on cognitive functioning. Both BD and cannabis use are independently associated with cognitive and functional impairments across different domains (Burggren et al., 2019; Ellingson et al., 2021; Martino et al., 2011; Post et al., 2003; Torres et al., 2011), making them suitable groups to illustrate NEUPSA’s potential range of detection. This report is not a comprehensive validation of the NEUPSA, instead serves to introduce the instrument and its application in a clinical context. We aimed to: 1) apply the NEUPSA in a clinical and healthy population, 2) compare NEUPSA domain sub-scores by against performance in standardized cognitive assessments, and 3) determine whether NEUPSA scores differed between healthy controls and participants with bipolar disorder and/or cannabis use to gather initial insight into its sensitivity to cognitive effects of such conditions. For this third aim, we hypothesized that amongst the non-CU groups, BD participants would have lower scores than healthy comparison (HC) participants, but cannabis use in BD participants would be associated with similar NEUPSA scores to HC participants, based on our prior work within this cohort (Miranda et al., 2025).

## Materials and methods

### The UCSD performance-based skills assessment-2 (UPSA-2)

The UPSA-2 uses role-play situations across six domains to evaluate functional capacity: comprehension/planning, finance, communication, transportation, household management, and medication management (Patterson et al., 2001). Comprehension and planning skills are assessed by having participants read a fictional article about a theme park, then describe the activities at the park and select items required to visit the park. Finance skills are assessed by having participants count change and describe paying for a fictional utility bill. Communication skills are assessed by having participants use an unplugged telephone to complete tasks such as rescheduling a medical appointment. Transportation skills are assessed by having participants read and interpret a bus route and plan a trip. Household management skills are assessed by having participants shop through a mock pantry using a shopping list. The medication management skills are assessed by asking participants to plan out a complex medication routine using four different drugs over one day.

### Development of the NEUPSA

The NEUPSA follows the tradition of assessing quantified process information to compliment traditional functional assessments, a concept similarly applied in the Wechsler Adult Intelligence Scale-Revised (Kaplan, 1995). Our group has also applied a similar approach to quantifying the cognitive processes that underlie performance on the Category Test (Minassian et al., 2003). Cognitive domains were selected based on established neuropsychological constructs known to underlie real-world functional performance: memory, attention, inhibition/impulsivity, speed of information processing, and executive function. These were chosen since better cognitive performance within these domains have been linked to higher functional ability, including those measured in the UPSA-2, in several clinical populations (Clark et al., 2022; Flores et al., 2022; Mahmood et al., 2018; Roye et al., 2022; Van Patten et al., 2022). Performance on specific UPSA-2 tasks was mapped to these domains through consensus discussion among three neuropsychology experts who have extensive experience in both cognitive assessment and functional capacity measurement. This mapping process ensured that each observed behavior during the UPSA-2 was anchored to a defined cognitive construct and that domain-level scoring would reflect theoretically relevant cognitive processes. For example, during the Transportation section of the UPSA participants are given points each time they correctly indicate locations on the maps and identify bus routes to specific locations at specific times when prompted by the test administrator. Using the NEUPSA scoring criteria, points are awarded for behaviors that correspond to relevant cognitive domains as follows: identification of trolley locations on a map within 30 seconds corresponds to speed of information processing, indicating when a bus will reach a destination after accounting for stops corresponds to executive functioning, reading the bus schedule for the correct day corresponds to attention, and waiting for the examiner’s prompts to be completed before responding corresponds to inhibition.

Scoring thresholds and range values were decided by consensus during pilot scoring, with the aim of balancing sensitivity to subtle errors and feasibility during administration. For simplicity and ease of scoring, all are either scored dichotomously or use graded scales (e.g., 0–2, 0–4, or 0–2–4) to capture varying degrees of accuracy or behavioral control. For example, broader scoring ranges were implemented for successful inhibitory control in the final two UPSA tasks as it has been observed that inhibitory control may diminish over time (Habay et al., 2026; Nigg, 2016; Tucha et al., 2017). The highest score (4) on these items reflects that the participant was able to exercise inhibitory control at the end of the task. The NEUPSA scoring sheet can be found in the Supplementary Data File.

Trained raters complete an additional structured scoring protocol during UPSA-2 administration (e.g., timing of task completion), capturing behavioral indicators across the five cognitive domains. To simplify scoring for each participant, a standardized Excel scoring sheet was used to calculate NEUPSA scores from UPSA-2 scores and the additional NEUPSA data collected. A total NEUPSA score is derived by summing domain scores, with higher scores indicating better cognitive performance. To minimize potential rater bias, the primary NEUPSA scorer was blinded to participants’ diagnostic and cannabis use status, and scoring sheets were then secondarily reviewed by another team member for accuracy. Incongruencies in scoring were resolved by discussion with the study investigator (i.e., trained neuropsychology expert who assisted in the development of the NEUPSA). NEUPSA scoring criteria are domain-specific and predefined to reduce subjectivity. Table 1 summarizes the NEUPSA domains, UPSA sections scored, and example behavioral indicators.

### Participants

This first application of the NEUPSA was conducted within a larger parent study on cognitive functioning in BD and cannabis use which occurred between August 2018-August 2022. Briefly, the parent study included a one-time in-person assessments of cognitive functioning and behavior over the course of approximately three hours. Assessments included five computerized tasks, one behavioral assessment, prepulse inhibition measurements, administration of the UPSA, and written self-report measures of symptoms and behavior (e.g., Hamilton Depression Rating Scale, Karolinska Sleepiness Scale). The convenience sample included the entire parent study cohort with available data; eighty-eight participants (18–50 years old) were recruited from the San Diego, CA area. Fifty were healthy comparison (HC) participants who had never met Structured Clinical Interview for DSM Disorders (SCID (RV/NP (Version 1.0.0) criteria for any Axis I psychiatric disorder and the remaining thirty-eight participants met SCID criteria for BD. Individuals with BD were clinically stable at testing (Young Mania Rating Score <20 and Hamilton Depression Scale [HAMD] ≤ 20). While this HAM-D level does not indicate complete symptom remission, it allowed inclusion of participants with mild-to--moderate residual symptoms to better represent real-world clinical populations. Participants were excluded for: (1) current alcohol or substance (excluding cannabis) dependence; (2) a history of neurological conditions, head trauma, or seizures; (3) treatment with electroconvulsive therapy; (4) stroke or myocardial infarction; (5) a positive urine toxicology result for non-prescribed oxycodone, phencyclidine, barbiturates, benzodiazepine, nortriptyline, buprenorphine, cocaine, amphetamine, methamphetamine, methadone, morphine, or THC (for non-cannabis using participants only); (7) active suicidality (assessed by the SCID and symptom ratings). All participants provided written informed consent to the current protocol approved by the UCSD Institutional Review Board.

Both HC and BD participants were classified by cannabis use status into two groupings: no cannabis use (CU-; less than 5× lifetime use and no use in the past 90 days) or used cannabis 4x or more use weekly for the past 90 days (CU+) (Haney et al., 2015; Henry et al., 2014; Papini et al., 2017; Ramaekers et al., 2009).

### Cognitive testing

As a part of the parent study, two cognitive tasks were employed on all participants to assess executive functioning, attention, inhibition and speed of information processing. These tasks were used to explore convergent validity with the NEUPSA. Due to the nature of the parent study, there was no comparator task available to assess memory.

The Iowa Gambling Task (IGT (Bechara et al., 1994)) was employed to assess risky decision making, an aspect of executive functioning. The IGT is a computerized task (~10 minutes) in which participants are instructed to select from 4 decks of cards (A, B, C, D) that yield hypothetical monetary rewards of various amounts at various levels of risk. After selecting a card, a participant wins a theoretical amount of money but may also lose some money. Decks A and B (risky choices) contain both large amounts of monetary gains, but also large losses compared to Decks C and D (safe choices), which contain smaller amounts of monetary gains but also smaller losses, making decks C and D “lower risk” and more advantageous over time. The IGT consists of 5 trial blocks (20 card selections per trial block) and scores are calculated per trial block and across all 5 trial blocks (total). The primary outcome measure was the total net difference score, calculated by subtracting the total number of risky choices from the total number of safe choices.

The 5 Choice-Continuous Performance Task (5C-CPT (Bhakta & Young, 2017; Pocuca et al., 2020)) was also employed to measure sustained attention, inhibition and speed of information processing. The 5C-CPT (~15 minutes) is a computerized cross-species neurocognitive task where participants are given a joystick and presented with 5 choices (white lines) in an arc on a computer screen. During “target trials,” a single circle appears next to one line. During “non-target trials,” all 5 circles appear next to every line simultaneously. Participants are instructed to move the joystick in the direction of the circle during a target trial or to not respond/move the joystick during a non-target trial. Some trials are “masked,” meaning that after the target(s) appear, they are quickly covered by a white bar, making them more difficult. For these analyses, we calculated outcome measures for only the unmasked trials.

5C-CPT outcome measures include d-prime (attention), false alarms (inhibition) and response reaction times (mean reaction time for correct responses; speed of information processing). Calculations for 5C-CPT outcome measures have previously been published by our group (Pocuca et al., 2020).

### Statistical analyses

Assumptions for equal variances (Levene’s or Box’s test of equality) and normality (Shapiro-Wilks test) were tested for IGT Net Difference scores, 5C-CPT scores and NEUPSA scores; variance and normality were tested across the entire sample and within each group. While IGT scores and NEUPSA scores (with the exception of Memory scores) were normally distributed, 5C-CPT scores were not; as such, non-parametric testing was employed as described below. Potential outliers were assessed using boxplots and Tukey’s method. Participants with missing cognitive data were excluded from analyses.

ANOVA or Chi-square tests were used to determine demographic differences between the four groups (HC/CU−, HC/CU+, BD/CU−, BD/CU+). Clinical and demographic covariates that differed between groups and/or were correlated with outcome variables (i.e., age and mania symptoms) were considered as covariates. Sensitivity analyses were conducted to determine the effects of these factors on outcome variables; the results remained consistent therefore we did not include covariates for these analyses.

To assess Aim 2 (compare NEUPSA domain sub-scores by against performance in standardized cognitive assessments), Spearman’s or Pearson’s correlational analyses were used to evaluate associations between NEUPSA domains and comparator task performance. Chi-square tests were used to evaluate group differences between categorical variables (NEUPSA Inhibition/Impulsivity scores and 5C-CPT false alarms).

To assess Aim 3 (test whether NEUPSA criteria and scoring can be used to detect effects of cannabis use and BD), 2 × 2 ANOVAs were conducted on NEUPSA scores with BD and cannabis use status as between-subjects factors. Cohen’s d or η^2^ effect sizes were calculated for main effects and interactions. Planned comparison t-tests were conducted between the HC/CU− group and the other groups, as well as within the BD group by CU status. Given our a priori hypotheses, the alpha level was set at 0.05. As NEUPSA Memory scores were not normally distributed, we used non-parametric Kruskal-Wallis tests and planned pairwise Mann-Whitney U tests for group comparisons. All statistical analyses were performed using SPSS 28.0 (Chicago, IL, USA).

## Results

### Cohort demographics

The sample groups consistently mostly of young, Caucasian adults (Mean age = 32) who had some higher education (mean = 14.75 years). The cohort was evenly distributed between males (46%) and females (53%). There were no significant differences between the four comparison groups in terms of sex or ethnicity distribution. There were also no significant differences in years of education between groups. The BD/CU− group was significantly older compared to the BD/CU+ and HC/CU+ groups, however sensitivity analyses revealed that including age as a covariate did not significantly impact the results. Demographic information, clinical data and total UPSA scores are presented in Table 2.

### NEUPSA scores positively correlate with cognitive task scores

In this first application of the NEUPSA, domain scores demonstrated meaningful associations with established cognitive measures. Higher executive function scores were associated with better decision-making on the Iowa Gambling Task (*r* = 0.27, *p* = 0.01). Attention scores correlated with both sustained attention hit rate (*r*_s_ = 0.24, *p* = 0.03) and d-prime sensitivity (*r*_s_ = 0.24, *p* = 0.03) on the 5 C-CPT. Faster processing speed scores trended toward shorter correct-response reaction times (*r*_s_ = −0.20, *p* = 0.06). In contrast, inhibition scores were not significantly associated with 5C-CPT false alarms (*χ*^2^ = 0.39, *p* = 0.71). Most participants (60%) demonstrated perfect inhibition on both measures, while 29% did so on the 5C-CPT only, 8% on the NEUPSA only, and 2% showed reduced inhibition on both.

### NEUPSA domain scores are associated with bipolar disorder and cannabis use

**Figure 1.**
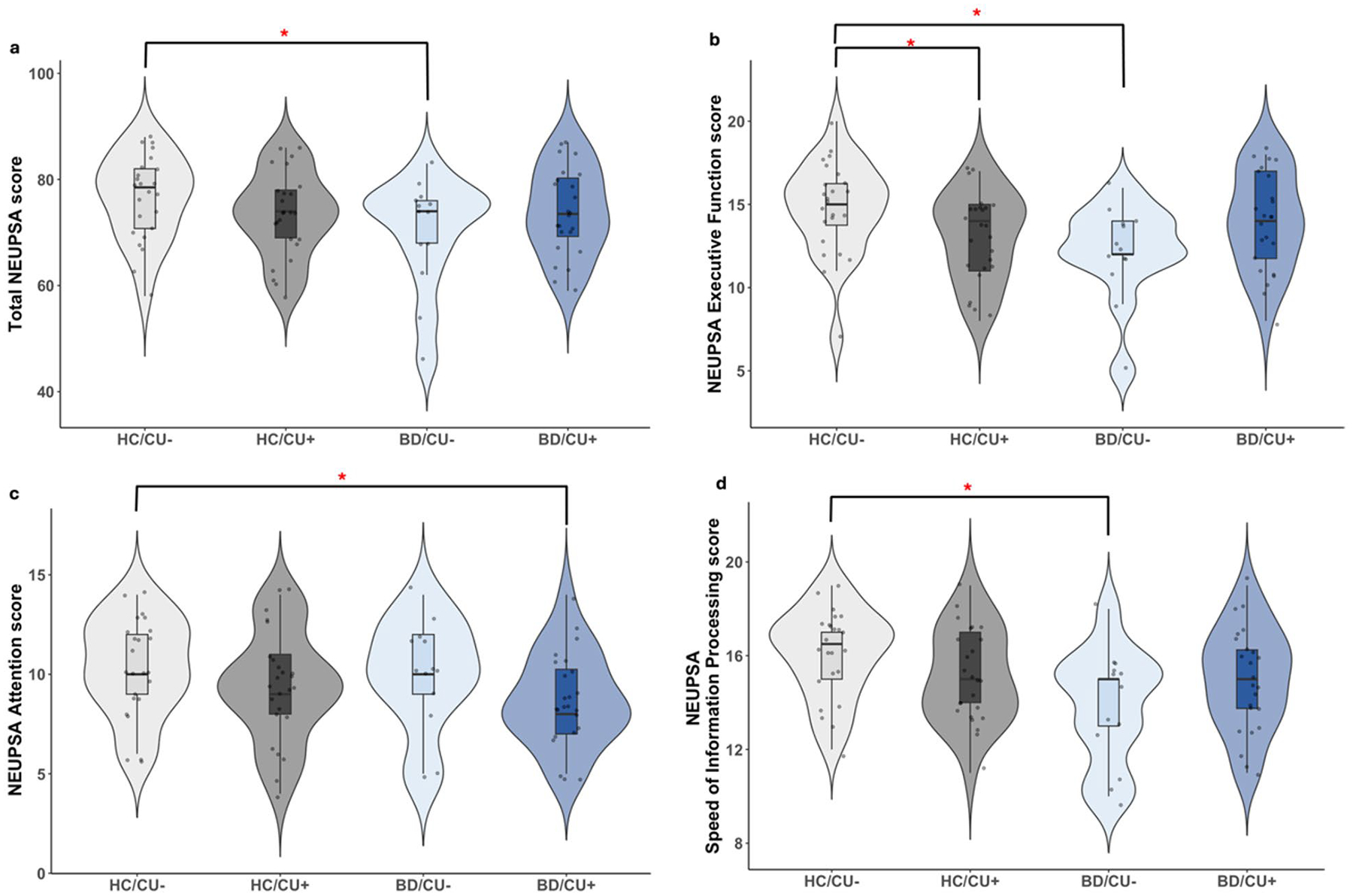
NeupsA scores are associated with cannabis use (cu) and bipolar disorder (Bd). a) total NeupsA scores were significantly lower in Bd participants compared to healthy comparison (hc) participants. b) NeupsA executive function scores were significantly lower in both hc/cu+ and Bd/cu− participants compared to hc/cu−. c) Bd/cu− participants had significantly lower NeupsA attention scores compared to hc/cu−. d) Bd/cu+ participants had significantly lower speed of information processing scores compared to hc/cu−. *denotes p < 0.05.

NEUPSA domain scores were associated with BD and cannabis use (Table 3). There was a trend interaction between BD and cannabis use on total NEUPSA score (*F*(1,83) = 3.4, *p* = 0.07, *η*^2^ = 0.04, Figure 1a) such that HC/CU− participants had significantly better NEUPSA performance compared to BD/CU− participants (*t* = 2.1, *p* < 0.05). However, there were no significant differences in total NEUPSA score between any other groups. In terms of executive function scores, there was a significant interaction between BD and cannabis use on executive function (*F*(1,83) = 7.70, *p* < 0.01, *η*^2^ = 0.09, Figure 1b). HC/CU− participants outperformed both their cannabis-using counterparts (*t* = 2.2, *p* = 0.03) and the BD/CU− participants (*t* = 2.7, *p* = 0.01) in the executive function domain. Interestingly, BD/CU+ participants performed comparably to HC/CU− participants (*t* = 1.01, *p* = 0.32) and trended toward higher executive function scores compared to BD/CU− participants (*t* = −1.8, *p* = 0.08).

There was no significant interaction between BD and cannabis use on attention, however cannabis use was linked to lower attention scores overall (*F*(1,83) = 3.96, *p* = 0.05, *η*^2^ = 0.05, Figure 1c). BD/CU+ participants had lower attention scores compared to HC/CU− participants (*t* = 2.42, *p* = 0.02). There was a significant interaction between BD and cannabis use (*F*(1,83) = 4.59, *p* = 0.04, *η*^2^ = 0.05, Figure 1d), and a significant main effect of BD on speed of information processing *F*(1,83) = 6.95, *p* = 0.01, *η*^2^ = 0.08). Processing speed was slower in BD/CU− participants relative to HC/CU− participants (*t* = 3.01, p < 0.01). There was also a near-significant difference between BD/CU+ participants (*t* = 1.85, *p* = 0.07), such that the BD/CU+ participants also had slower processing speed relative to the HC/CU− participants. Chi-square analyses on memory and inhibition/impulsivity NEUPSA scores did not reveal significant differences in proportions between the four comparison groups (Table 3).

### NEUPSA internal consistency

To assess the internal consistency of the NEUPSA, Cronbach’s α was calculated for each subscale (Table 4). Given that Cronbach’s alpha is influenced by item scoring and response formats, we examined the consistency within each subscale separately. The internal consistency estimates for memory (*α* = 0.69) and executive functioning (*α* = 0.67) approached the lower bound conventional threshold for acceptable reliability (i.e., 0.7(George & Mallery, 2003; Tavakol & Dennick, 2011)). However, the speed of information processing subscale (*α* = 0.57) exhibited poor reliability. Internal consistency estimates for attention and inhibition/impulsivity were not calculated given that heterogeneous scoring methods were used within these subscales leading to varied response scaling (e.g., 0–2, 0–4, 0–2–4).

## Discussion

In this report, we present the NEUPSA structured scoring system, compare NEUPSA scoring against validated cognitive assessments and offer preliminary evidence of the measure’s ability to capture meaningful cognitive variation among clinical groups. First, we described the development of NEUPSA, how it is designed to measure cognitive processes underlying performance in the UPSA-2, and defined its initial scoring criteria. We then provided preliminary evidence that the NEUPSA can meaningfully capture cognitive functioning by demonstrating that NEUPSA domain scores aligned with independent measures assessing similar constructs. Specifically, NEUPSA executive function scores correlated with Iowa Gambling Task performance, while NEUPSA attention and processing speed scores showed associations with 5C-CPT performance. Lastly, we demonstrated significant group differences in NEUPSA scoring in several cognitive domains between clinical groups (i.e., BD and CU) and healthy comparison adults. Participants with BD performed worse than healthy controls on executive function and processing speed, consistent with literature documenting cognitive slowing and executive dysfunction in BD (Bora, 2018; Ehrlich et al., 2022; Post et al., 2003). Cannabis use was associated with lower attention scores, with the effect most pronounced in BD/CU+ participants. BD/CU+ participants performed comparably to healthy controls on executive function, echoing prior findings that cannabis use in BD can be associated with better performance on some decision-making tasks (Miranda et al., 2025). Although the NEUPSA inhibition domain did not significantly differ when compared to the 5C-CPT, a larger proportion of participants’ inhibition errors were captured on only the 5C-CPT (72%) than only the NEUPSA (20%), potentially reflecting differences in cognitive demands between a naturalistic role-play environment and a rapid-response attentional task.

### Integration of cognitive process scoring into functional assessment

The NEUPSA builds on the UPSA by adding cognitive process scoring to an existing performance-based functional capacity measure. While the UPSA captures accuracy on ecologically valid tasks such as financial management, transportation, and medication management, it does not formally record how participants arrive at their responses. The NEUPSA addresses this gap by attempting to isolate and quantify the cognitive processes underlying the UPSA items (Kaplan, 1988). Similar methodology underlies other functional measures, such as the Complex Task Performance Assessment (CTPA (Wolf et al., 2017)), the Texas Functional Living Scale (TFLS (Cullum et al., 2001)), and the Virtual Reality Functional Capacity Assessment Tool (VRFCAT-SL (Keefe et al., 2016)). These tools demonstrate that process-oriented scoring can reveal subtle cognitive impairments missed by accuracy scores alone. The NEUPSA advances this tradition while minimizing additional administration burden by integrating cognitive scoring directly into the UPSA workflow.

Like the CTPA, TFLS, and VRFCAT-SL, the NEUPSA supports the broader observation that process scoring during functional tasks can uncover domain-specific cognitive weaknesses with ecological relevance. For example, the CTPA has been shown to detect deficits in cognitive flexibility and attentional control in Parkinson’s dementia (Davis et al., 2019), and the TFLS correlates strongly with global cognitive status in dementia populations (Cullum et al., 2001). The VRFCAT-SL has been linked to global neuropsychological performance in Parkinson’s disease (Turner et al., 2021). The NEUPSA’s distinction is that it adapts an already widely used functional tool rather than introducing a new assessment, making it potentially easier to implement in settings where the UPSA is already in use.

### Limitations

Several limitations warrant consideration. The sample was modest, particularly within BD subgroups, and derived from a parent study not specifically designed to validate NEUPSA. Although our analyses identified some statistical differences in NEUPSA scoring, BD participants within this cohort were clinically stable and did not exhibit substantial cognitive impairment, limiting our ability to detect meaningful specificity and sensitivity of the NEUPSA tool. This first version of the NEUPSA used mixed scoring scales (e.g., 0–1 vs. 0–4) without weighting, so domains with larger scoring ranges may have contributed disproportionately to total scores. Although scoring was conducted by trained staff with random accuracy checks, some raters were not blinded to group status. Comparator cognitive measures were limited, and no independent memory measure was available for validation. Correlations between NEUPSA and performance on the available comparator tasks were also modest, likely due to the limited range in NEUPSA scoring for this cohort. The findings should therefore be considered preliminary.

### Considerations for refinement

Future refinements may standardize scoring ranges to ensure equivalent item influence. Internal consistency was acceptable for memory and executive function but lower for other domains, and attention and inhibition/impulsivity were not evaluated due to heterogeneous scoring formats. Other refinements may include standardizing scoring scales, improving item clarity, and using factor analysis to confirm domain structure (Tavakol & Dennick, 2011). While test administrators did not report issues with using the NEUPSA criteria, and training in the NEUPSA framework was complementary to UPSA training, a thorough evaluation of feasibility is warranted to examine factors such as added time for administration and interpretation, compared to the UPSA alone. Establishing inter-rater reliability is also essential, especially if the measure is to be widely adopted in clinical or research practice. Although the NEUPSA is not designed to be a diagnostic tool, the sensitivity and specificity of the NEUPSA to detect cognitive impairment should also be examined. This refinement may be more useful in other clinical populations with established cognitive impairments, such as individuals with mild cognitive impairment or Alzheimer’s Disease-related dementias.

### Potential clinical and research applications

The NEUPSA’s main advantage lies in its efficiency and ecological validity. It allows clinicians to capture both functional capacity and cognitive process data within the same testing session, reducing the need for a separate neuropsychological battery when resources or patient tolerance are limited. Clinicians could use NEUPSA results to guide individualized cognitive remediation, identify specific targets such as processing speed or executive function, and monitor changes over time. In research, the NEUPSA may serve as an outcome measure in intervention trials, particularly those aimed at improving everyday functioning.

### Conclusion

This first clinical application of the NEUPSA demonstrates that integrating process-oriented cognitive scoring into an established functional capacity measure is both feasible and informative. Within a single administration, the NEUPSA revealed domain-specific cognitive strengths and weaknesses, particularly in executive function, attention, and processing speed, that were not apparent from total UPSA scores alone. By capturing how individuals approach real-world tasks, rather than relying solely on whether they complete them correctly, the NEUPSA offers a richer profile of functional ability with potential value for both clinical decision-making and research. These initial observations suggest that the NEUPSA could help clinicians identify cognitive targets for intervention, monitor changes over time, and bridge the gap between neuropsychological testing and everyday functioning. Further work is needed to refine the scoring system, establish reliability, and evaluate its utility across broader clinical populations.

## Supplementary Material

SupplementaryMaterial_NEUPSAscoring

Supplemental data for this article can be accessed online at https://doi.org/10.1080/23279095.2026.2676165.

## Figures and Tables

**Table 1.** Description of NEUPSA variables and scoring.

Domain	UPSA sections scored	Summary of items	Example errors	Max score
Memory				20
*Immediate recall*	Communication and Comprehension/Planning	Recall of essential details (e.g., directions, opening/closing times). Retention of specific information (e.g., names, telephone numbers). Memory for multiple activities	Forgetting to not eat before the role-play medical appointment.	16
*Recognition memory*	Medication management	Recall over multiple instances	Taking a medication (e.g., “Linophen”) with another medication (e.g.“BRB”).	4
Executive function	Comprehension/Planning, Financial skills, Transportation, Shopping, Medication management	Planning and organizing Problem-solving Information retrieval Temporal reasoning Decision-making in a practical context Adherence to guidelines/Following instructions	Being unable to list seven items that are important to bring to the fictional water park.	20
Speed of information processing	Financial skills, Transportation, shopping and Medication management	Rapid calculation Quick identification of transportation options Efficiency in task completion	Providing the incorrect amount for $1.02 in coins in under 1 minute.	20
Attention	Comprehension/Planning, Financial skills, Communication, Transportation and shopping	Focus and concentration Sustained attention Engagement and responsiveness	Uses a dollar bill instead of coins when prompted to provide tester with $1.02 in coins.	20
Inhibition/Impulsivity	Comprehension/Planning, Financial skills, Communication, Transportation and medication management	Self-regulation Impulse control Direction following	Interacts with the phone before any verbal instructions are presented in the communication skills portion of the UPSA-2.	20

**Table 2.** Demographic information for participants.

Mean (SD)	A	B	C	D	Group differences
	HC/CU− (*n* = 24)	HC/CU+ (*n* = 26)	BD/CU− (*n* = 14)	BD/CU+ (*n* = 25)	
Age	34.4(9.4)	30.4(8.1)	39.1(8.3)	29.5(8.3)	C > D, B F(3,85)=4.3 *p* = 0.007
Education (years)	15.8(2.8)	14.6(2.1)	14.7 (2.2)	14.0(2.4)	Ns *F*(3,85)=2.3
Gender					ns; χ^2^=4.2
Male	13	11	5	9	
Female	11	13	8	14	
Non-binary			1	2	
Trans		2			
Race/Ethnicity					ns; χ^2^=10.9
Caucasian	16	16	7	9	
African-American	2	2	1	4	
Hispanic	3	4	3	9	
Asian	3	2	2	1	
Additional groups		2	1	2	
Bipolar type					
BD I			10	21	
BDII			1	4	
Other			3		
YMRS	2.9(2.3)	4.0(3.4)	6.4(4.3)	5.6(3.2)	C, D > A *F*(3,85) = 4.6 *p* = 0.005
HAM-D	4.0(2.7)	5.7(3.8)	8.9(5.1)	7.8(4.7)	C, D > A *F*(3,85) = 5.9 *p* = 0.001
UPSA total score	100.4(9.45)	96.7(10.5)	91.0(11.2)	98.4(10.3)	Ns *F*(3,83) = 2.0 *p* = 0.1

Data are mean (standard deviation) or counts.

**Table 3.** Total NeupsA score and sub-scores for each group.

NEUPsA score	A	B	C	D	Group differences
	HC/CU−	HC/CU+	BD/CU−	BD/CU+	
Total	76.6(7.9)	73.5(7.8)	70.1(10.5)	73.9(8.23)	A > C
Executive function	14.8(2.8)	13.2(2.6)	12.2(2.8)	14.0(2.9)	A > B, C
Attention	10.4(2.4)	9.4(2.7)	10.0(2.8)	8.7(2.3)	A > D
Speed of information processing	16.1(1.9)	15.2(2.0)	13.9(2.4)	15.0(2.2)	A > C
Memory	16.1(2.9)	16.3(2.5)	15.7(3.5)	16.9(2.0)	ns; KW = 0.8
Immediate recall	12.3(2.7)	12.4(2.5)	12.2(3.7)	13.1(1.9)	ns; KW = 1.2
Recognition	3.9(0.4)	3.9(0.3)	3.5(1.1)	3.8(0.4)	ns; KW = 1.8
Impulsivity/Inhibition (% score = 20/20)	63%	77%	58%	71%	ns; χ^2^=0.6

Data are presented as means (standard deviations), or percentages.

**Table 4.** Internal consistency of the NeupsA (cronbach’s α) for each NeupsA subscale.

Domain	*a*
Memory	0.69
*Immediate recall*	0.69
*Recognition memory*	0.60
Executive function	0.67
Speed of information processing	0.57
Attention	–
Inhibition/Impulsivity	–
