# Supplementary material for: A process-oriented functional capacity assessment: First application of the neuropsychological evaluation of the UPSA (NEUPSA)
Source: Appl Neuropsychol Adult. Author manuscript; Available in PMC 2026 Jun 18. (PMC13277714; doi:10.1080/23279095.2026.2676165)
Supplement: SupplementaryMaterial_NEUPSAscoring [file NIHMS2185108-supplement-SupplementaryMaterial_NEUPSAscoring.pdf]

Neuropsychological Evaluation from the UCSD Performance-Based Skills Assessment (NEUPSA)

Subject ID:

A) MEMORY: Score 1 point for each correct item for a total maximum score of 20. Items 1-16 constitute immediate recall; 17-20 represent recognition memory.

1) Comprehension/Planning

Q1: How to arrive: ______

Q2: Time Open: ______

Q3: Time Close: ______

Q4: Activity 1: ______

Q5: Activity 2: ______

Q6: Activity 3: ______

Q7: Activity 4: ______

2) Communication skills

Q28: Address inquiry name of person ______

Q28: Address inquiry name of town ______

Q28: Address inquiry name of person ______

Q29: Telephone number ______

Q35: Item 1 to bring to appointment ______

Q36: Item 2 to bring to appointment ______

Q37: Blood draw - no food ______

Q37: Blood draw - no liquids ______

Q37: Blood draw - can drink water ______

Q37: Blood draw - 12 hour interval ______

3) Medication Management

Q54: Linophen taken without BRB 1st time ______

Q54: Linophen taken without BRB 2nd time ______

Q54: Linophen taken without BRB 3rd time ______
Q54: Linophen taken without BRB 4th time ______

Immediate Recall subscore (0-16): ____

Recognition Memory subscore (0-4): ____

TOTAL MEMORY DOMAIN SCORE (0-20): ____

B) EXECUTIVE FUNCTION: Score 1 point for each correct item for a total maximum score of 20.

1) Comprehension/Planning

Q8: Item 1 to bring to water park: ______
Q9: Item 2 to bring to water park: ______
Q10: Item 3 to bring to water park: ______
Q11: Item 4 to bring to water park: ______
Q12: Item 5 to bring to water park: ______
Q13: Item 6 to bring to water park: ______
Q14: Item 7 to bring to water park: ______

2) Financial skills

Q18: Correct change from $10 ______
Q22: Explain new balance on bill ______

3) Communication skills

Q32: Provide current appointment for voicemail ______
Q33: Provide new appointment for voicemail ______

Q34: Provide telephone number for voicemail ______

4) Transportation

Q44: How long to wait for bus ______
Q45: When bus will reach destination ______
Q46: When to catch bus for appointment ______

5) Shopping

Q47-50: Identifies all four items to purchase ______

6) Medication Management

Q51: Parlenol taken correctly ______
Q52: BRB taken correctly ______
Q53: Cyclomeovan taken correctly ______
Q54: Linophen taken correctly ______

TOTAL EXECUTIVE FUNCTION SCORE (0-20): ____

C) SPEED OF INFORMATION PROCESSING: Score 1 point for each item completed Correct within the specified period for a total maximum score of 20. Record time for each response. Note incorrect response reaction time in parentheses ()

2) Financial skills

	Score	Time (seconds)
Q15: Correct amount for $1.02 in coins (1 minute)	______	___________
Q16: Correct amount for $6.73 (45 seconds)	______	___________
Q17: Correct amount for $12.49 (45 seconds)	______	___________
Q18: Correct change from $10.00 (1 minute)	______	___________
Q19: Identify utility company name (15 seconds)	______	___________
Q20: Identify amount of payment due (30 seconds)	______	___________
Q21: Identify current charges due (30 seconds)	______	___________

Q23: Identify due date (30 seconds) ______ ______
Q24: Identify account number (30 seconds) ______ ______
Q25: Identify telephone number to call (30 seconds) ______ ______

4) Transportation

Q38: Identify bus (30 seconds) ______ ______
Q30: Identify bus fare (30 seconds) ______ ______
Q40: Identify number to call (30 seconds) ______ ______
Q41-42: Identify location of trolleys (30 seconds) ______ ______
Q43: Identify location Bus #54 (30 seconds) ______ ______
Q44: How long to wait for bus (1 minute) ______ ______
Q45: When bus will reach destination (1 minute) ______ ______
Q46: When to catch bus for appointment (1 minute) ______ ______

5) Shopping

Q47-50: Shopping Task completed in 5 minutes ______ ______

6) Medication Management

Q51-54: Medication task completed in 15 minutes ______ ______

TOTAL SPEED OF INFORMATION PROCESSING SCORE (0-20): ____

SUM OF REACTION TIME FOR ALL CORRECT RESPONSES Q15-Q46 ____

AVERAGE REACTION TIME FOR ALL CORRECT RESPONSES Q15-Q46 ____

D**) ATTENTION**: Score for each appropriate response up to a total maximum score of 20. Errors, incorrect responses, or repeated prompts are given a 0 score. If no initial incorrect responses occur in a domain, score as 2; other successfully completed items are scored as 1.

1) Comprehension/Planning

Q1-7: Incorrect initial response for any item ______

Q4-7: Subject names activities from their experience, not article ______

Q8-14: Subject lists more or fewer than 7 items ______

2) Financial skills

Q15-18: Incorrect initial response for any item ______

Q15: Uses dollar bill instead of coins ______

Q15-17: Requests repetition of prompts ______

Q18: Requests repetition of prompt for change task ______

3) Communication skills

Q26-37: Incorrect initial response for any item ______

Q26-37: Requests repetition of prompts ______

Q30-33: Does not attend to voicemail instruction, interacts instead ______

4) Transportation

Q38-46: Incorrect initial response for any item ______

Q44-46: Reads schedule for incorrect day ______

Q44-46: Requires repetition of prompts ______

5)Shopping

Q47-50: Search task completed with time limits (<5 minutes) ______

Q47-50: Initial incorrect responses (scratched out answers) ______

TOTAL ATTENTION SCORE (0-20): ____

E) INHIBITION/IMPULSIVITY: Score for each appropriate response up to a total maximum score of 20.If the subject engages in the behavior listed (e.g., premature response), give them a 0 score. Participants who do not exhibit disinhibited/impulsive behavior receive a score of 2 for Comprehension, Financial, and Communication domain items, and 4 points each for the Transportation and Medication domains.

<u>1) Comprehension/Planning</u>

Q1-14: Verbal premature responding or responses without prompt
(e.g., answers question about when the water park closes before it is asked) ______
Q1-14: Interacts with binder without instructions -
attempts to look at other pages following the water park article ______

<u>2) Financial skills</u>

Q15-18: Subject touches or moves coins/bills without instruction ______
Q19-25: Premature responses to questions on utility bill ______

<u>3) Communication skills</u>

Q26-37: Subject interacts with (touches/moves) phone when first ______
placed in front of them before any verbal instructions are presented
Q26-37: Premature responses (subject picks up phone and starts ______
dialing before instructions are finished)

<u>4) Transportation</u>

Q38-46: Premature responses to prompts, e.g., interrupts examiner ______
to answer questions

<u>6) Medication Management</u>

Q51-54: Subject begins task before instructions are finished ______

TOTAL INHIBITION/IMPULSIVITY SCORE (0-20): ______

NEUPSA Summary Scoring Worksheet

**Domain**	**Domain Score**	
Memory:	____________	(0 to 20)
Executive Function	____________	(0 to 20)
Speed of Information Processing	____________	(0 to 20)
Attention	____________	(0 to 20)
Inhibition/Impulsivity	____________	(0 to 20)
**TOTAL NEUPSA** Score	____________	**(0 to 100)**
